# Which trap is best? Alternatives to outdoor human landing catches for malaria vector surveillance: a meta-analysis

**DOI:** 10.1186/s12936-022-04332-1

**Published:** 2022-12-09

**Authors:** Jordan Eckert, Seun Oladipupo, Yifan Wang, Shanshan Jiang, Vivek Patil, Benjamin A. McKenzie, Neil F. Lobo, Sarah Zohdy

**Affiliations:** 1 Department of Mathematics and Statistics, Auburn University, 221 Parker Hall, Auburn, AL 36849 USA; 2 Department of Entomology and Plant Pathology, Auburn University, Auburn, AL USA; 3 Department of Biosystems Engineering, Auburn University, Auburn, AL USA; 4 Geospatial Research, Analysis and Services Program, Centers for Disease Control and Prevention, Atlanta, GA USA; 5 Eck Institute for Global Health, University of Notre Dame, Notre Dame, IN USA; 6 College of Forestry, Wildlife, and Environment, Auburn University, Auburn, AL USA; 7 US President’s Malaria Initiative, Centers for Disease Control and Prevention, Atlanta, GA USA; 8 Molecular Biophysics and Biochemistry, Yale University, New Haven, CT USA

**Keywords:** *Anopheles*, Collection, HLC, Meta-analysis, Mosquito

## Abstract

**Background:**

Human landing catches (HLC) are an entomological collection technique in which humans are used as attractants to capture medically relevant host-seeking mosquitoes. The use of this method has been a topic of extensive debate for decades mainly due to ethical concerns. Many alternatives to HLC have been proposed; however, no quantitative review and meta-analysis comparing HLC to outdoor alternative trapping methods has been conducted.

**Methods:**

A total of 58 comparisons across 12 countries were identified. We conducted a meta-analysis comparing the standardized mean difference of *Anopheles* captured by HLC and alternative traps. To explain heterogeneity, three moderators were chosen for analysis: trap type, location of study, and species captured. A meta-regression was fit to understand how the linear combination of moderators helped in explaining heterogeneity. The possibility of biased results due to publication bias was also explored.

**Results:**

Random-effects meta-analysis showed no statistically significant difference in the mean difference of *Anopheles* collected. Moderator analysis was conducted to determine the effects of trap type, geographical location of study, and the species of *Anopheles* captured. On average, tent-based traps captured significantly more *Anopheles* than outdoor HLC (95% CI: [− .9065, − 0.0544]), alternative traps in Africa captured on average more mosquitoes than outdoor HLC (95% CI: [− 2.8750, − 0.0294]), and alternative traps overall captured significantly more Anopheles gambiae s.l. than outdoor HLC (95% CI: [− 4.4613, − 0.2473]) on average. Meta-regression showed that up to 55.77% of the total heterogeneity found can be explained by a linear combination of the three moderators and the interaction between trap type and species. Subset analysis on An. gambiae s.l. showed that light traps specifically captured on average more of this species than HLC (95% CI: [− 18.3751, − 1.0629]). Publication bias likely exists. With 59.65% of studies reporting p-values less than 0.025, we believe there is an over representation in the literature of results indicating that alternative traps are superior to outdoor HLC.

**Conclusions:**

Currently, there is no consensus on a single “magic bullet” alternative to outdoor HLC. The diversity of many alternative trap comparisons restricts potential metrics for comparisons to outdoor HLC. Further standardization and specific question-driven trap evaluations that consider target vector species and the vector control landscape are needed to allow for robust meta-analyses with less heterogeneity and to develop data-driven decision-making tools for malaria vector surveillance and control.

## Background

The accurate understanding and quantification of the drivers of vector distribution and pathogen trans- mission could be critical for effective vector control. For mosquitoes, a number of environmental and human-associated factors shape pathogen transmission dynamics [1, 2]. These transmission parameters describe the relationship between entomological indicators (such as abundance, feeding behaviour, longevity) and epidemiological outcomes [3]. Thus, the first step in understanding the dynamics between mosquitoes and mosquito-borne diseases is the estimation of these parameters. Of these, entomological indicators that are a result of mosquito sampling/collection are the most pertinent. This is because collection methods are used to gather baseline information on mosquito abundance, diversity, distribution, biting frequency and behaviour, mosquito survival, and infection rates [1, 4]. Taken together, the synthesis of such key information is crucial for planning optimal mosquito intervention strategies.

The suitability of a mosquito collection method is species-specific and should be coupled with sampling methods that take advantage of specific behaviours. While there are about 4000 species of mosquito described today [5], only a few genera, such as *Aedes* and *Anopheles*, are efficient transmitters of human pathogens. Notably, *Anopheles* spp. transmit the parasites responsible for human malaria. Malaria is the most serious arthropod-vector borne disease causing morbidity and mortality in humans. To date, perhaps the greatest success recorded in the fight against malaria has been through the use of mosquito control interventions such as insecticide- treated nets (ITNs) and indoor residual spraying (IRS) that target specific biting and resting behaviours of *Anopheles* spp. adult females [6–8], behaviours that were identified through mosquito surveillance methods. Thus, mosquito surveillance methods that provide information about behaviours are essential for understanding parasite transmission dynamics and the impact of vector control tools. For example, one key metric in understanding malaria transmission dynamics is the entomological inoculation rate (EIR), this indicator takes into account *Anopheles* biting rate and the proportion of mosquitoes carrying infectious *Plasmodium* sporozoites. By combining this information, an estimate of the number of infectious bites an individual will get over a set period of time can be calculated and this value is often used as a key indicator of transmission. Data on vector bionomics gathered through *Anopheles* collection methods can also be used to develop and implement targeted vector control interventions that exploit specific *Anopheles* behaviours. In addition, the influence of socioenvironmental conditions on currently employed *Anopheles* surveillance methods should be studied [9, 10].

To date, several methods have been employed in the estimation of mosquito biting behaviours [10–14], however, human landing catches (HLC) have been suggested as the gold standard for malaria vector surveillance, being widely used and the most direct way to measure *Anopheles* vector biting on humans. HLC is a method of mosquito collection that uses humans and their natural production of carbon dioxide (CO2), heat, and odour as bait to capture host-seeking mosquitoes. HLC is an important collection method because it uses humans as an attractant to determine *Anopheles* abundance over a set period and human biting rate, the crucial metric that when combined with data on presence of infective human *Plasmodium* sporozoites gives the EIR. By using humans as baits, HLC facilitate the collection of human-biting mosquitoes capable of transmitting human malaria para- sites (*Plasmodium* spp.). By directly measuring biting hourly, HLC can also be used to determine peak human biting times and quantify differences in indoor/outdoor biting behaviours [15–17].

Despite the widespread use of HLC as an arguably unparalleled method in determining host seeking and biting behaviours relevant for understanding *Anopheles* vector exposure and human *Plasmodium* parasite transmission, the use of HLC has long been a topic of controversy. HLC requires collectors to stay awake during overnight collections, and although collectors may be provided with prophylaxis to protect them from malaria infection [18], they may be exposed to other vector- borne pathogens such as the causative agents of lymphatic filariasis, chikungunya, leishmaniasis, among other agents [16]. HLC also require expertise from both col- lectors and supervisors and are physically demanding, requiring collectors to work throughout the night [19]. The results obtained through HLC, and some other trap- ping tools, are also heavily influenced by the attractiveness of the human collector to the *Anopheles* species [20, 21]. Furthermore, HLC typically provides data on only mosquitoes that feed on human legs [16, 17] possibly ignoring populations that obtain a blood meal from other parts of the human body.

In Africa, as elsewhere, alternatives to HLC, many of which can measure human exposure and biting and therefore provide a proxy for EIR estimations have been proposed and evaluated under various conditions for the collection of *Anopheles* species. For example, varying designs of light traps including the Centers for Disease Control (CDC) light trap [22, 23] odour baited-traps [24, 25] electrocution traps [16, 26], decoy traps [27], tent traps [28, 29], barrier screens [30], and a combination of these traps [31] have all been explored as alternatives. Several of these alternative collection methods have been conducted in direct comparison with HLC with differing results [12, 20, 32–36]. For example, one study comparing methods showed CDC miniature light traps captured at least twice the number of *Anopheles* captured by HLC [37]. These comparative studies only use male volunteers, as the ethical concerns of using women and children are too great, even though they are the most vulnerable. Perhaps what is often overlooked, for the efficiency of a trap type, is the sensitivity and correlation between host-seeking/resting behaviour and malaria-pathogen transmission. In this respect, a trap would be appropriate if it collects representative populations (or species) of adult females (fed and unfed). Such trap data would provide essential information on Anopheles abundance, human biting rate (HBR), and EIR. In recent years several programmes have stopped HLC or had discussions about halting the use of HLC for various reasons including risk of exposure to other vector-borne diseases [16]. Therefore, a comparable and effective collection method is needed for malaria vector surveillance. Indoors, CDC light traps have been used as an alternative to indoor HLC where an individual sleeps under a bed net and acts as an attractant towards the light trap, with conversion factors being developed for EIR; however, there is no consensus on outdoor mosquito collection alternatives to HLC for use in malaria surveillance, despite a large number of individual field studies comparing alternative traps to outdoor HLC. To date, no systematic review or meta-analysis combining these results has been conducted.

Consequently, the aim of this literature review and meta-analysis is to compare mean differences in capture rates between alternative outdoor mosquito collection methods for malaria surveillance and outdoor HLC, to determine which tools could be used to replace outdoor HLC, and to examine variation in the literature and the effects of geography, general trap type, trap bias, and target species on collection results. Specifically, this study aimed to address whether publication bias, geographical location of the comparison study, species composition, and trap type (light trap, tent trap, electrocuting box trap), and categorical classification (biological, physical, chemical) had significant effects on the alternative trapping methods outdoors and their comparability to outdoor HLC.

## Methods

### Literature search, inclusion criteria, and study selection

Preferred Reporting Items of Systematic reviews and Meta-Analyses (PRISMA) recommendations were followed for the literature search, creating the inclusion criteria, and data extraction [38]. Databases were searched independently during May 2020. Searching was done by a group of four researchers (SO, YW, SJ, VP) using (“human landing catches”) AND ((“human landing catches alternatives”) OR (“HLC”) OR (“vector surveillance”)) as keywords. Keywords were employed respectively and the search results were combined using advanced search tools. No language or date restrictions were set. The specific databases searched were left up to the discretion of the researcher with the only requirement being that each investigator searched three separate databases. A total of five databases were accessed (BASE, PubMed, Web of Science, Google Scholar, and Science.gov). After the removal of duplicates, there were 944 items left. For the next step, the paper titles and abstracts were screened by splitting into two groups (Group 1: JE, SZ, YW; Group 2: SO, SJ, VP, BM). Group 1 screened the first 472 papers and group 2 screened the last 472 sorted alphabetically by author’s last name. Each member of the group voted on the eligibility of each paper. For Group 2, tiebreakers were included as eligible. A paper was eligible for inclusion if it received a majority vote as per the inclusion criteria. The inclusion criteria were:

- A paper must be an entomological malaria surveillance experiment
- Outdoor HLC must have been performed
- The study must have involved an alternative trapping method
- The outcome of interest in the meta-analysis was the standardized mean difference between outdoor HLC and alternative traps. Therefore, the study must have recorded mean *Anopheles* captured per trap over a defined period or a similar metric/way of calculation.
- At least one *Anopheles* mosquito must have been captured by both outdoor HLC. Similarly, at least one *Anopheles* mosquito must have been captured by the comparative alternative method.

**Fig. 1:**
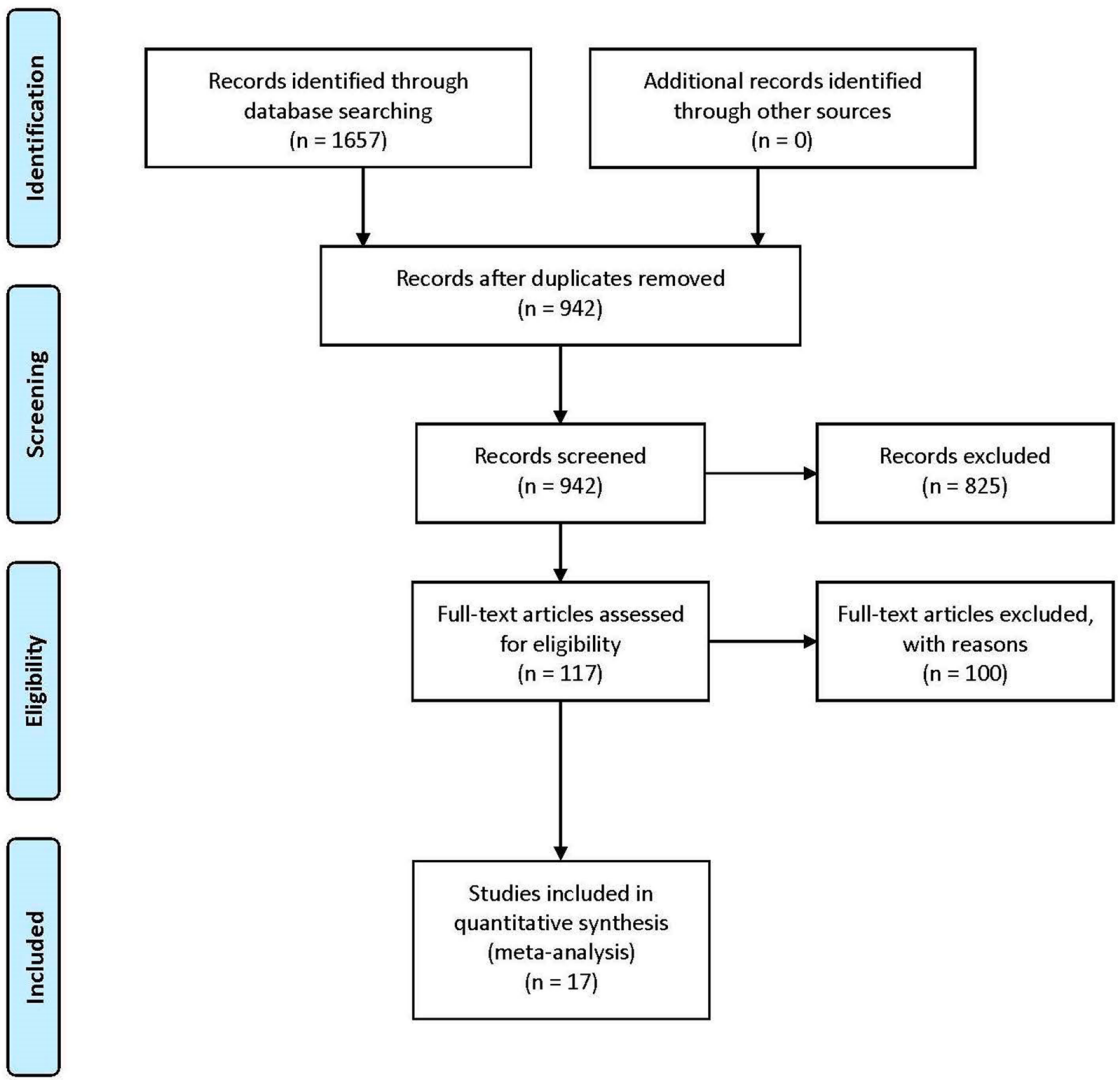
PRISMA guidelines were used for study selection and inclusion for the meta-analysis

Acceptance for publication was also taken as a criterion for inclusion. No grey literature or conference abstracts were included. Data from a total of 17 articles were extracted yielding a total of 58 comparisons of alternative traps to outdoor HLC. Figure 1 shows the PRISMA flow diagram. While all efforts were made to include eligible papers, some papers might have failed to be included due to limitations with search terms; papers with publication dates after the search period are failed to be included as well. Publications included for analysis were [11, 19, 24, 39–52].

### Data extraction and preparation

Two researchers extracted the data (JE, SO). Any discrepancies were resolved by the lead author after revisiting the articles. The following variables were extracted from selected articles:

- **Author:** author(s) of the included study
- **Year:** included study publication year
- **Country:** country the experiment was performed in
- **Coordinates:** exact coordinates of experiment site if given in the included study. If testing was done at multiple sites or coordinates were not given, approximations were used
- **Trap name:** name of trap being tested
- **Species name:** name of species captured and identified during the experiment
- **Species:** categorical variable of captured species into one of three groups. The species categories were:
  - ‘*Anopheles gambiae*’ – species belonging to *An. gambiae* species complex
  - ‘*Anopheles funestus*’—species belonging to *An. funestus* group
  - ‘*Anopheles* spp.’—all other species not belonging to *An. gambiae s.l*. or *An. funestus s.l.*
- **Length:** the number of days collections were conducted.
  Three additional variables were created:
- **Category:** categorical variable for the category classification of alternative trapping methods as defined in [20]. The type categories were:
  - ‘Biological’
  - ‘Chemical’
  - ‘Physical’
  - ‘Physical/Chemical’
- **Type:** categorical variable for the classification of alternative trapping meth ods. The type categories were:
  - ‘Tent’
  - ‘Light’
  - ‘Electrocuting’
  - ‘Other—Mechanical’
  - ‘Other—Passive’
- **Africa:** categorical variable equal to 1 if the experiment was conducted in Africa or equal to 0 if the experiment was conducted outside of Africa. Africa was the only region with enough studies to be used as a moderator in analysis with enough statistical power.

**Fig. 2:**
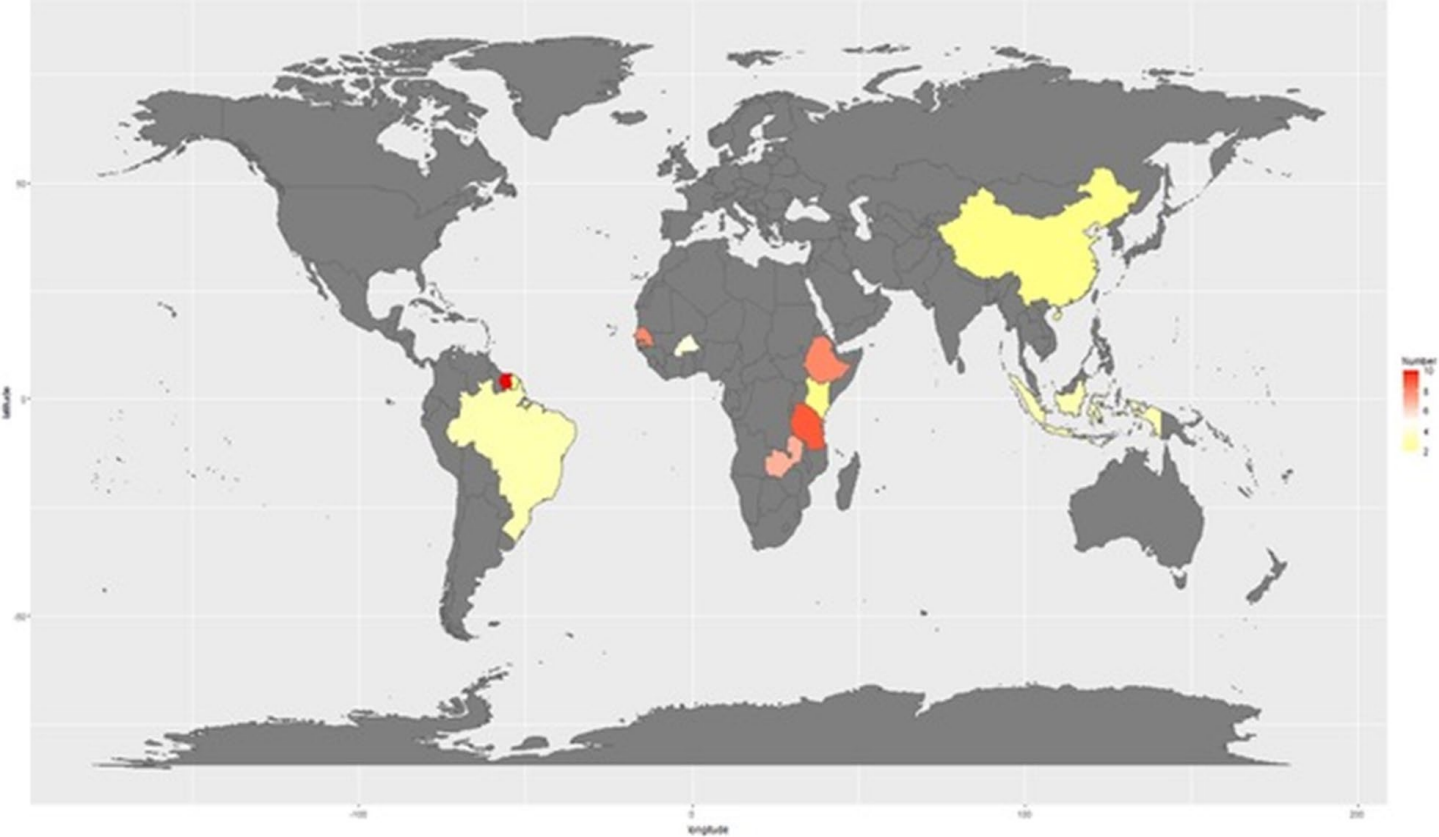
Study locations were distributed around the world, although most studies were conducted in the African continent. Heat map coloration indicates the number of studies in each location with darker colors indicating a higher number of studies. The complete list of studies are available on https://github.com/JordanEckert/malaria

Publication dates of included studies ranged from 1995 to 2019. There were 28 different alternative traps included in the dataset. A total of thirty-one *Anopheles* species were represented in the meta-analysis. Four articles had experiments from South America, two from Asia, thirteen from Africa, and one from Oceania. This difference in experimental location was the reason behind creating a moderator for Africa, as opposed to a specific country or region. Across all included papers, there were a total of twelve unique countries (Fig. 2).

If necessary, data were extracted from graphics using R version 3.6.3 [53] and the *metaDigitise* [54] package version 1.0.1. For articles that included multiple com- parisons to HLC, the individual comparisons were added. Treatment effect sizes and standard errors were calculated using *esc* [55] package version 0.5.1 and were recorded during data extraction.

### Statistical analysis

Meta-analytic techniques were conducted using *metafor* [56] package version 2.4-0, *meta* [57] package version 4.11-0, and R. Some functions of the *dmetar* [58] package version 0.0.9 were also used in analysis which required installation from Github. The standardized mean difference (“Hedges’ g”) of mosquitoes captured in the two methods was used as the effect size. Effect sizes were calculated with the control being the outdoor HLC; negative effect sizes indicated that outdoor HLC captured fewer mosquitoes than the alternative method. Mosquitoes captured were chosen as the outcome variable due to capture numbers being universally available across HLC and all alternative trapping methods. A random effects framework was used for the modeling to account for heterogeneity. *τ*^2^, the variance of the distribution for the true effect size under such a framework was estimated using a restricted maximum likelihood (REML) approach. The model framework chosen assumes that the observed estimates of treatment effect can vary across studies because of real differences in the treatment effect in each study as well as sampling variability. The point estimate for an individual study then assumes that there is a second source of error that is hierarchical and that the observed effect sizes of a study deviate from their true value because of the sampling error. All random-effects models used the Hartung-Knapp adjustment for the variance of the pooled effects estimator. Moderator analysis was also performed with all moderators being considered random effects during their respective moderator analysis.

Outlier detection was done using the *find.outliers()* function in the *dmetar* package. The approach to classifying a study as an outlier was a brute force approach wherein an included study for which the upper bound of the 95% confidence interval was lower than the lower bound of the pooled effect confidence interval was considered an outlier, or similarly for when the lower bound of the 95% confidence interval was higher than the upper bound of the pooled effect confidence interval. The method described above is not comprehensive for finding outliers; it is possible that outliers existed that were not considered.

Multi-model inference was done using the *multimodel.inference()* function in R, wherein all possible combinations of the Type, Africa, and Species variables with their respective interactions were fitted in a meta-regression. Model selection was based on having the lowest corrected Akaike Information Criterion (AIC). Resampling methods were used to validate the robustness of the meta-regression. The standard in meta-analysis is to use permutation testing [59]. Using *metafor*’s built in *permutest()* function, one thousand iterations were run.

## Results

### Random-effect meta-analysis

Analysis performed on fifty-eight comparisons alternative traps to outdoor HLC yielded a Hedges’ g value of *g* = − 0.8544. *I*^2^ as a measure of heterogeneity was 98*.*3% which is quantified as substantial heterogeneity (Table 1). It is likely the meta-analysis results lack the ability to detect significant mean difference due to heterogeneity. High heterogeneity can be potentially caused by a single study with an anomalous effect size. Outlier analysis was performed to attempt to explain if the heterogeneity was caused by extreme points or other underlying factors. Of the original 58 comparisons, only 36 were synthesized after outlier removal. Substantial heterogeneity was still found after outlier removal, indicating that other sources might be contributing in tandem (Table 2). Using moderator analysis and meta-regression, an attempt was made to better explain the statistical heterogeneity present and quantify it.

**Table 1:** Random-effect Meta-Analysis

n	Hedge’s g	95% Confidence Interval	*τ*^2^	*I*^2^
58	− 0.8544	[− 1.751, 0.0562]	10.6943	98.3%

Results reveal that there is no statistically significant difference between alternative trapping methods and HLC in terms of total *Anopheles* collected

**Table 2:** Random-effect Meta-Analysis with Outlier Removed

n	Hedge’s g	95% Confidence Interval	*τ*^2^	*I*^2^
36	− 0.5905	[− 0.7574, − 0.4235]	0.1618	78.6%

Results show that alternative trapping methods collected significantly more *Anopheles* mosquitoes than HLC

### Explaining heterogeneity

#### Meta-regression

**Fig. 3:**
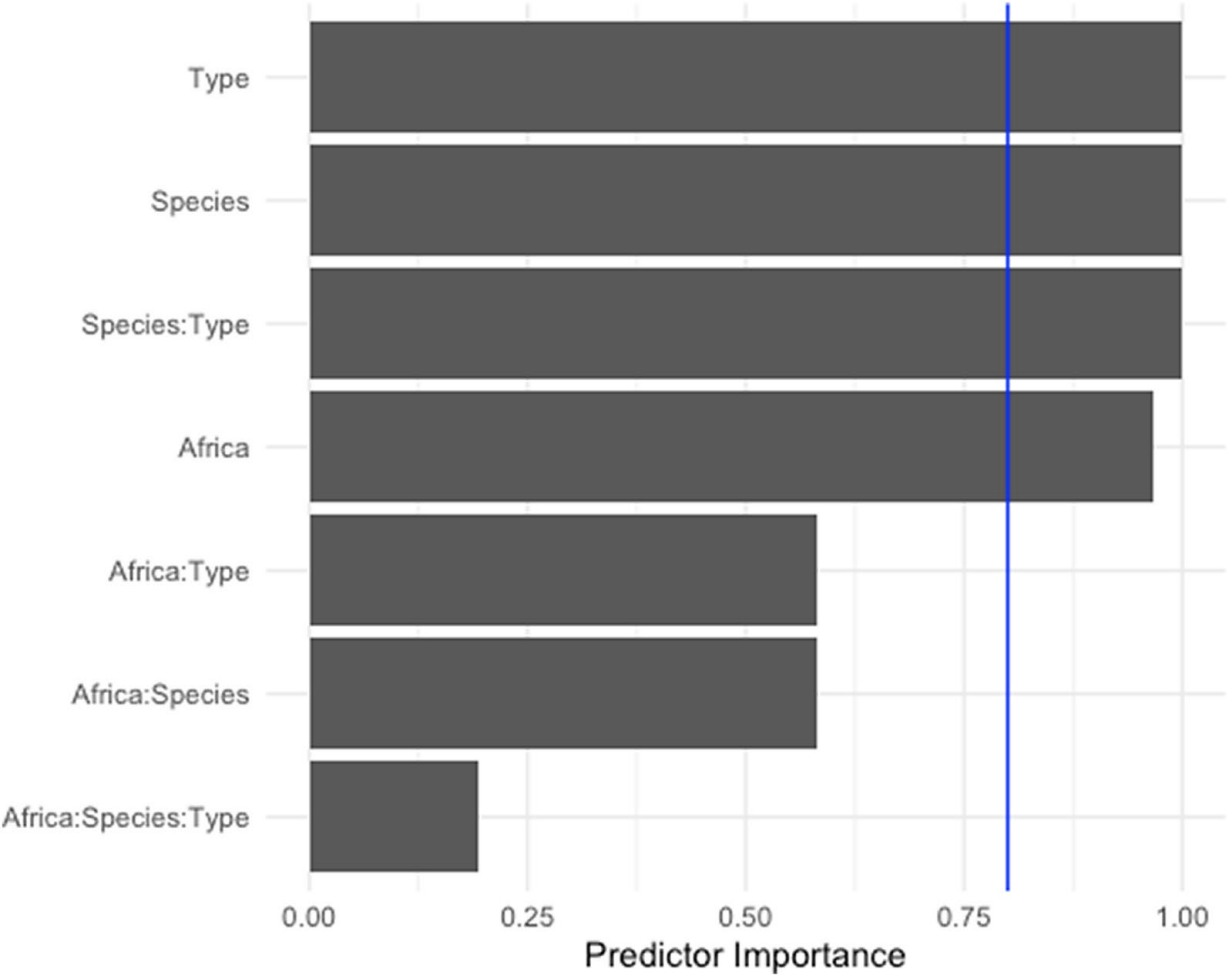
Using multimodal inference, importance for the model’s fit shows that individual ‘Type’, ‘Species’, ‘Africa’ and the interaction of ‘Type’ and ‘Species’ variables meet the threshold and are classified as important variables to be included in the final model

Meta-regression was performed to see if the statistical heterogeneity could be explained using a linear combination of moderators instead of individual associations. The top regression model based on the AIC criterion included Type, Species, Africa, and the interaction of Type and Species (Fig. 3). Figure 3 shows the modelled average predictor importance plot. The fitted meta-regression model reported *R*^2^ = 55*.*77% which, in the context of meta-regression, implies that the linear combination of these four variables explains 55*.*77% of the heterogeneity present.

#### Moderator analysis

Three separate moderator analyses were performed wherein the random-effects meta-analysis framework was re-fit under observations being grouped into categories. Moderator analysis provides the framework to see between and within group heterogeneity. Some groups had to be combined into larger groups so that there was enough power to detect significant results. The first moderator analysis used the ‘Type’ variable to generate the groupings. There were four groups for this analysis—tent traps, light traps, a combined group of traps that were neither tent nor light but still used passive methods for collection, and another combined group of traps that were neither tent nor light but used mechanical methods for collection. The second moderator analysis used the ‘Africa’ variable to generate the groupings. There were only two groups for this analysis—studies performed in Africa and studies not performed in Africa. The third moderator analysis used the ‘Species’ variable to generate the groupings. There were three groups for this analysis—*Anopheles gambiae* complex, *An. funestus* group, and *Anopheles* spp. An additional moderator analysis looking at trap ‘Categories’ was also conducted, but due to the subjective nature of categorization those results are not included here (see Annex for trap category moderator findings).

**Fig. 4:**
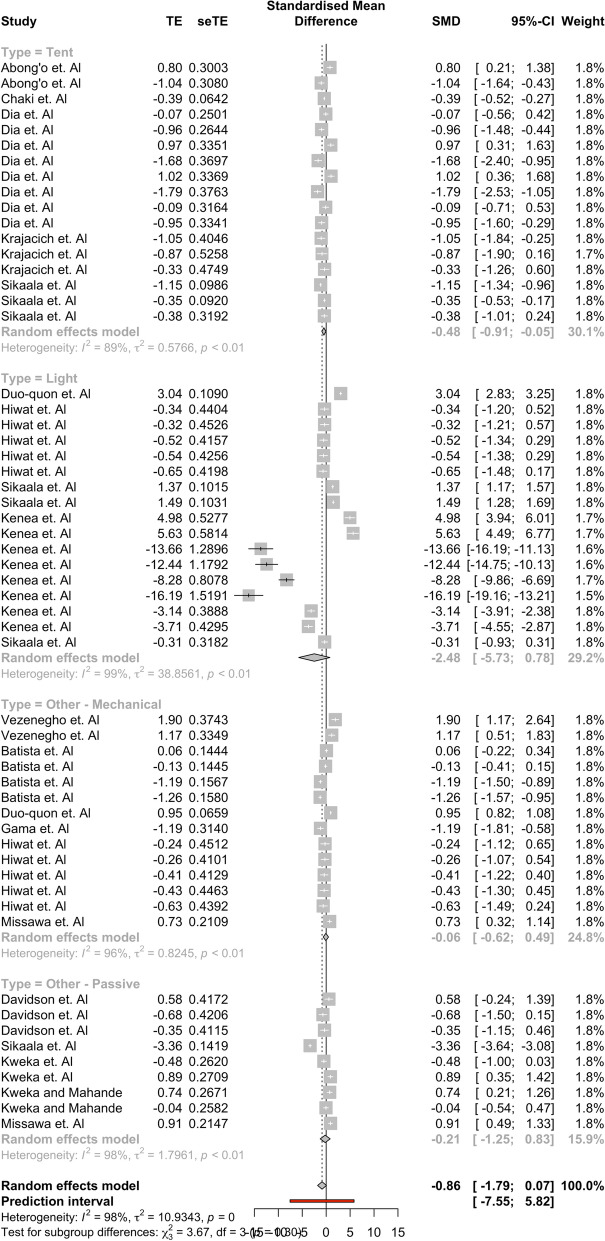
A forest plot broken into the four ‘Type’ groups. The plot shows the 95% confidence interval for each study and their respective weights. Most observations seem to fall within a similar range with the exception of four observations found in the Kenea et al. paper

**Fig. 5:**
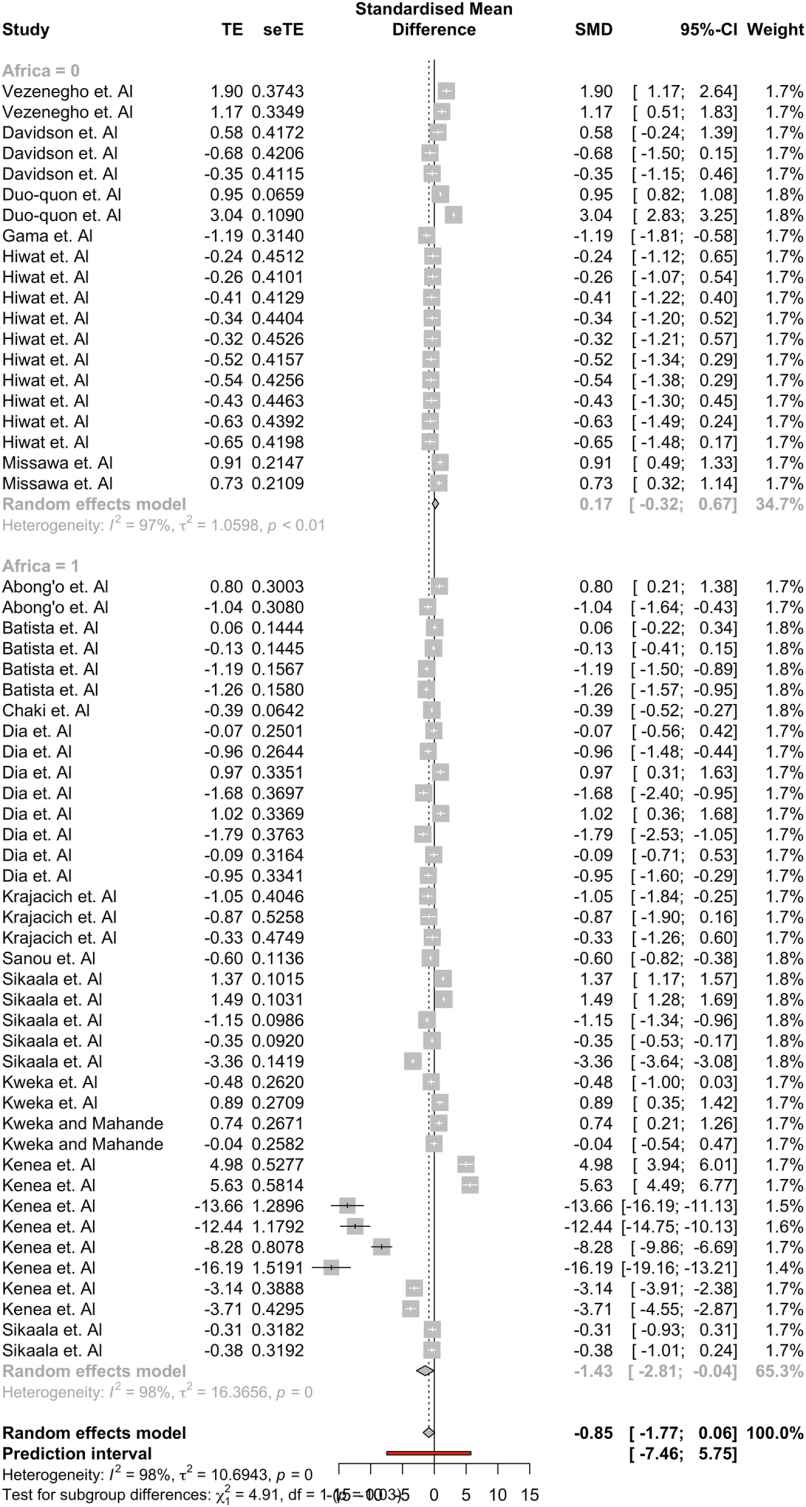
A forest plot broken into the two ‘Africa’ groups. The plot shows the 95% confidence interval for each study and their respective weights. Most observations seem to fall within a similar range with the exception of four observations found in the Kenea et al. paper

**Fig. 6:**
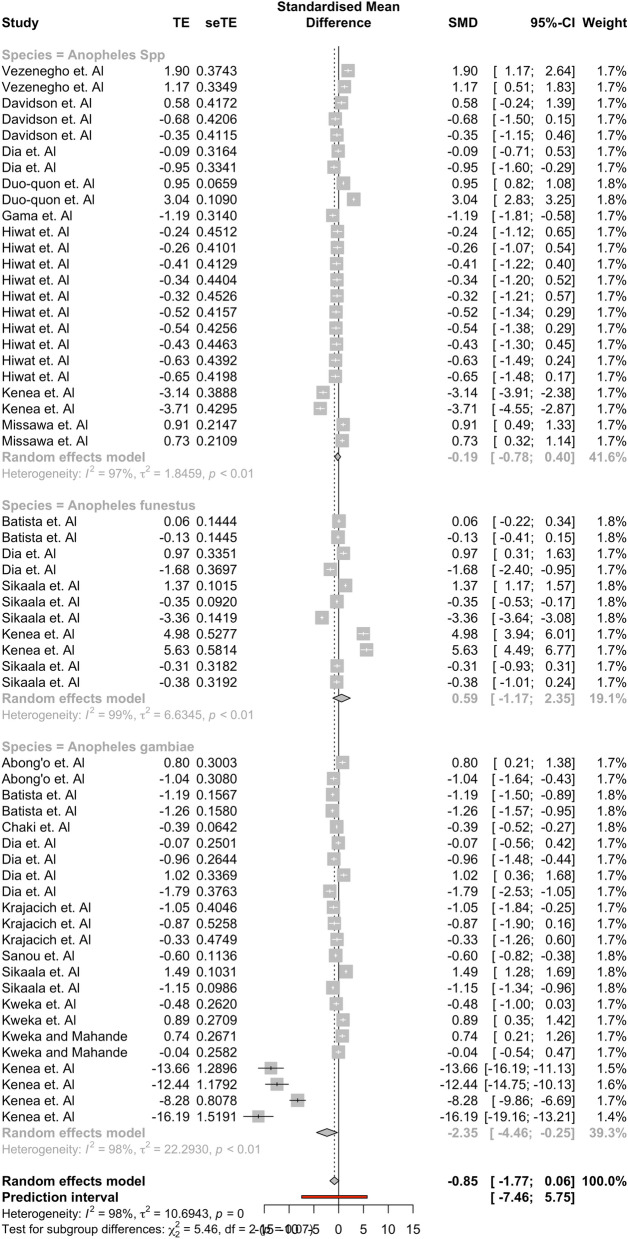
A forest plot broken into the three ‘Species’ groups. The plot shows the 95% confidence interval for each study and their respective weights. Most observations seem to fall within a similar range with the exception of four observations found in the Kenea et al. paper

Results showed that the tent group captured a significantly higher mean difference of *Anopheles* compared to outdoor HLC (95% CI: [− 0.9065, − 0*.*0544]). No other ‘type’ group had a significant mean difference between the alternative traps and outdoor HLC, indicating that there was no statistical difference between the mean capture numbers of these alternative trap groups and outdoor HLC. Significant results were found in the full comparisons of studies performed on the Africa subgroup ([− 2.8750, − 0.0294]) and the An. gambiae subgroup ([− 4.6475, − 0.2330]) in their separate, respective moderator analyses as well. None of the other 95% confidence intervals in these respective moderator groups found indicated that outdoor HLC collected on average more mosquitoes than their respective moderator groups. Results for each moderator analysis are found in Tables 3, 4, 5. Forest plots showing the effect sizes and confidence intervals within specific studies are found in Figs. 4, 5, 6. The assumption that there was not a common estimate of *τ*^2^ across subgroups was made for each analyses. For robustness, the results were computed under a change of assumption so that there was a common estimate of *τ*^2^ across subgroups. No changes to statistical significance were detected for any group between the two assumptions.

**Table 3:** Subgroup Analysis using ‘Type’

	n	Hedge’s g	95% confidence interval	*Τ*^2^	*I*^*2*^
Tent	17	− 0.4805	[− 0.9065, − 0.0544]	0*.*5766	88*.*9%
Light	17	− 2.4770	[− 5.7332, 0.7792]	38*.*8561	98*.*8%
Other—Passive	14	− 0.0650	[− 0.6150, 0.4851]	0*.*8245	96*.*4%
Other—Mechanical	9	* − *0*.*2083	[*− *1*.*2493*,* 0*.*8327]	1*.*7961	98*.*3%

Subgroup analysis shows that tent traps capture significantly more *Anopheles* mosquitoes than other trap types. Only one electrocuting trap study was included in this, so it was removed for analysis

**Table 4:** Subgroup Analysis using ‘Africa’

	n	Hedge’s g	95% confidence interval	*Τ*^2^	*I*^*2*^
Studies not in Africa	20	0.1748	[− 0.3243, 0.6738]	1.0598	96.5%
Studies performed in Africa	38	− 1.4268	[− 2.8095, − 0.0440]	16.3656	98.2%

Subgroup analysis shows that alternative trapping methods performed in Africa capture significantly more *Anopheles* mosquitoes than HLC

**Table 5:** Subgroup analysis using ‘Species’

	n	Hedge’s g	95% confidence interval	*Τ*^2^	*I*^*2*^
*An. gambiae* complex	23	− 2.3543	[− 4.4613, − 0.2473]	22.2930	97.7%
*An. funestus* group	11	0.5875	[− 1.1705, 2.3455]	6.6345	99.0%
*Anopheles* spp.	24	− 0.1930	[− 0.7835, 0.3975]	1.8459	97.2%

Subgroup analysis shows that alternative trapping methods capture significantly more *Anopheles gambiae* than HLC

#### Analysis of subsets

Of the total 57 comparisons synthesized, 22 of the comparisons were from captures involving *An. gambiae s.l.* and 11 were *An. funestus s.l.*, with the rest being other *Anopheles* spp. A separate moderator analysis using ‘Type’ was done independently for both *An. gambiae * (Table 6). *s.l*. and *An. funestus s.l.* (Table 7). For the *An. gambiae s.l.* subset, the light trap type had a statistically significant higher mean difference of *An. gambiae s.l.* caught when compared to outdoor HLC (95% CI: [− 18.3751, −  1.0629]).

**Table 6:** Subgroup analysis using ‘Type’ for *Anopheles gambiae*

	n	Hedge’s g	95% confidence interval	*Τ*^2^	*I*^2^
Tent	11	− 0.5231	[− 1.0929, 0.0468]	0.6146	90.6%
Light	5	− 9.7190	[− 18.3751, − 1.0629]	47.9384	99.3%
Other	6	− 0.2434	[− 1.2149, 0.7282]	0.8077	94.5%

Subgroup analysis using the ‘Type’ moderator on the subset of *Anopheles gambiae s.l.* shows that traps that incorporate light capture significantly more mosquitoes than HLC. The ‘Other’ group was collapsed into a single group to increase statistical power for analysis

**Table 7:** Subgroup analysis using ‘Type’ for *Anopheles funestus*

	n	Hedge’s g	95% confidence interval	*Τ*^2^	*I*^2^
Tent	4	− 0.3539	[− 2.0478, 1.3399]	1.0098	89.5%
Light	4	2.8827	[− 1.6527, 7.4181]	7.9393	97.6%
Other	3	− 1.1428	[− 5.9189, 3.6333]	3.6761	99.4%

Subgroup analysis using the ‘Type’ moderator on the subset of *Anopheles funestus s.l.* shows that no trap type had significant results when compared to HLC. The ‘Other’ group was collapsed into a single group to increase statistical power for analysis, however, the number of synthesized studies for each group was below the traditional threshold of *n* ≥ 5 limit. More synthesized studies are required for definitive analysis

#### Publication bias

**Fig. 7:**
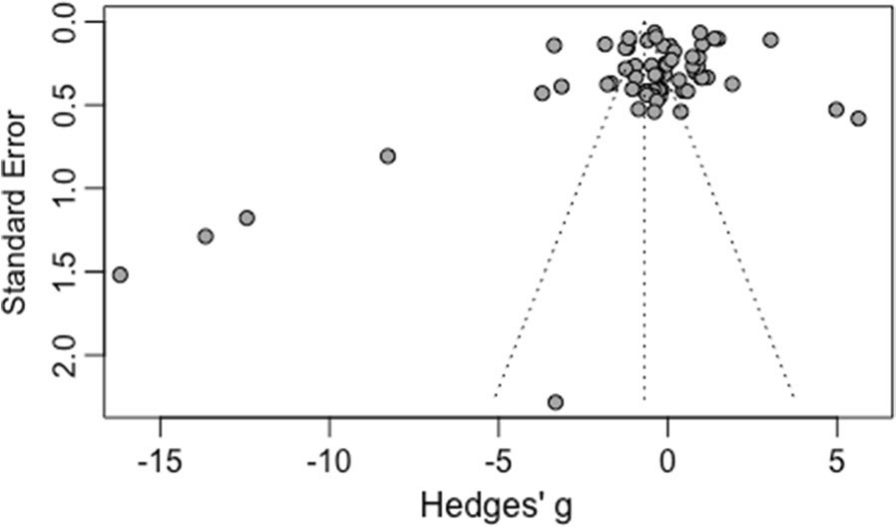
A funnel plot of the standard errors versus effect size. Each study was created to examine publication; the studies should follow the outlined funnel shape if publication bias is not present. However, this figure shows that there is publication bias in this meta-analysis

An unfortunate weakness of any meta-analysis is the lack of ability to include all available data. The two most likely causes for not being able to include all available data are missing papers during searching, and the “file-drawer” problem i.e., many results that are not statistically significant are more likely to not become published [54, 56, 57]. The funnel plot of the data wherein each comparison’s standard error is plotted against the effect size is represented in Fig. 7. One would typically expect the data to follow the prescribed funnel shape if publication bias was not present. However, the figure shows a large grouping at the top of the funnel with some points landing well outside the funnel—a deviation from the typical shape. Further testing for publication bias was done using the Egger’s test of asymmetry (see Table 8 for results). The graphical representation of the data with the p-value for the t-test of the intercept was not significant (*p*^∗^ = 0.0508). This value, while not statistically significant, has a lot of practical significance- especially when coupled with the oddly shaped funnel plot. Given the closeness of the Egger’s test, funnel plot, and the trim-and-fill analysis (Table 9) the authors believe that it is reasonable to assume some moderate level of publication bias is present in the analysis. It is likely that certain entomological surveys that showed either no difference between the deployed trapping method and outdoor HLC, or even results where HLC performed better than the deployed method, were not published depending on the objective of the survey. Not including these results could be potentially biasing the results presented towards significance for alternative trapping methods in each of the mode.

**Table 8:** Egger’s Test for Asymmetry

Egger’s Test	Intercept	95% confidence interval	t	Pr(>|t|)
	− 3.179	[− 6.3, − 0.06]	− 1.997	0.05081156

The Egger’s test was performed on the funnel plot to test for asymmetry, which would be a sign of publication bias. At a 95% confidence level, the closeness of the p-value and shape of the corresponding funnel plot indicate that publication bias likely exists

**Table 9:** Trim and fill analysis

	Hedge’s g	95% confidence interval	*Τ*^2^	*I*^*2*^
Random effects model	0.5506	[− 0.5077, 1.6088]	19.9866	98.7%
Prediction interval		[− 8.4165, 9.5176]		

A total of 19 studies were added to compensate for the possible funnel plot asymmetries. The additional studies show that the pooled effect is underestimated in the original meta-analysis

## Discussion

This meta-analysis aimed to compare the mean difference of *Anopheles* mosquitoes captured by outdoor human landing catches comparative to alternative trapping methods. The initial pooled results showed substantial heterogeneity in the literature and no clear evidence that one alternative trap is best to replace outdoor HLC. As a follow-up three different moderator analyses were used to explain heterogeneity. When examining trap type as a moderator, tent traps in particular collected an overall higher average number of *Anopheles* than HLC. Since the majority of studies comparing HLC and alternative traps were conducted in Africa, moderator analysis was conducted comparing studies in Africa with those conducted elsewhere in the world. It is possible that additional studies showing HLC collecting more mosquitoes than alternative traps in Africa may have been conducted, but not published. Similarly, papers might not have shown up in the search or been outside of the range of search dates or inclusion criteria. When examining species as a moderator, the results indicated that in general, alternative traps collected more *An. gambiae s.l.* than HLC. A meta-regression showed that over 55% of the heterogeneity can be explained by a linear combination of these three variables and the interaction of Type and Species variables. Subset analysis the *An. gambiae s.l.* shows that light traps capture on average more mosquitoes than HLC. Results for the *An. funestus s.l.* did not have enough statistical power to detect significant differences, if they truly exist.

### Limitations

Many of the results presented in this meta-analysis showed no significant difference in the mean number of *Anopheles* captured by outdoor HLC and alternative methods. To determine if this was truly equivalency and not just a lack of evidence, statistical methods such as two one-sided test analyses should be conducted. If equivalency is determined, no conversion factor would be needed. A major limitation in this approach is that the trap comparisons are based on the total number of *Anopheles* collected/night, and accurately estimating human biting risk remains a challenge. Alternative methods may collect more *Anopheles* than HLC, but this does not mean that they more accurately estimate biting risk. Therefore, if alter- native approaches are used to replace HLC, correction factors may be necessary to estimate biting risk for the calculation of EIR. However, a precise conversion factor for metrics such as EIR may not be necessary. While EIR is a very valuable entomological indicator, these values are dynamic and absolute EIR numbers may not be necessary when a relative EIR could suffice to inform vector control decisions. Future work testing for equivalency will provide additional information, but the quality of collections not just the quantity of collections should be considered in future work. Additionally, to further understand the malaria vector landscape, a metric could be developed to show how HLC and alternative traps perform in the context of vector control interventions. Issues arise if the relationship between HLC and trapping methods is not a monotonic relationship—such as linear. The importance of the interaction of species and trap type in the meta-regression implies that the idea of a single “calibration” factor that can be broadly translated across ecological contexts is unlikely.

It has been reported that CDC light traps in a rice irrigation area had a bias towards younger *An. arabiensis* mosquitoes as indicated by parity dissections [60]. Such parity is not considered in this analysis, but could be incorporated in future work to get a full picture of the landscape. If an individual study had multiple comparisons to outdoor HLC, each individual comparison was added. It is possible that by adding these individual comparisons in such a manner, the results could have an over representation of certain locations where alternative traps do better. Future work should try to incorporate a more robust selection of spatial variables and their subsequent interactions to help calculate the true spatial effect on heterogeneity.

It is worth reiterating that while all efforts were made to include eligible papers, some papers might have failed to be included due to limitations with search terms, such as papers with publication dates after the search period. For example, [61] show that new double net traps and human odor baited CDC-LT caught more female *An. arabiensis* than traditional CDC-LT; however, this was published in 2020 and was not included due to publication after the search period. Future meta-analyses could include information on female and male trapping collection rates for both alternative traps and outdoor HLC.

Many comparative studies are recording data over the course of several months to even years. Temporal variables such as collection during the rainy and dry season are difficult to incorporate under the current reporting methods, and so were not used during this meta-analysis. Such variables could play a large role in further explaining heterogeneity. Changing the reporting standard to include data from collections in publications would allow for the interaction of temporal variables and alternative trapping methods to be observed.

No prior knowledge of the known differences between the *An. gambiae* subspecies was incorporated into analysis. *Anopheles arabiensis* readily feed on cattle [62]. By assuming that all feeding behaviours of *An. gambiae* subspecies are the same, it biases the results towards the alternative methods by collecting vectors that more do not as readily feed on humans, not to mention that humans have a specific close, medium, and long-range attraction to mosquitoes that is often difficult to replicate. Future research endeavours should look at including feeding behaviours of the *An. gambiae* subspecies, if there is enough readily available data. In that same vein, more studies on trap types that specifically capture *An. funestus s.l.* are necessary to statistically determine which trap type has the best potential for capture, if one exists. It is possible that additional studies showing HLC collecting more *An. funestus s.l.* than alternative traps may have been conducted, but not published.

In addition to ethical considerations, there are also biases (known but mostly uncharacterized) that may influence HLC’s data. Therefore, the use of HLC must be either critically examined and understood or alternatives to HLC that ablate these biases should be sought. For example, collector bias may impact the number and quality of collections from HLC as individuals may have differing degrees of mosquito attraction, although this may be addressed to some extent with appropriate study design. Additionally, HLC collectors who are recruited and trained are mostly men between the ages of 20–50, while the populations most vulnerable to malaria are women and children. Vector control interventions are often selected for implementation based on data from HLC collections; however, the possibility of differential attraction between men and women and children may not be adequately considered. Alternative methods that can be used to address this bias may provide a broader understanding of vector bionomics.

To date, despite claims that HLC may put collectors at risk for infection with vector-borne pathogens, there is only one study that examined the safety risks of HLC for collectors, and this study focused exclusively on the risk of malaria [18]. In this work, the authors showed that when HLC collectors were provided with malaria prophylaxis, malaria incidence was lower than in non HLC collectors. However, no published study has reported the risk of mosquito collectors being exposed to other arthropod vectors or vector-borne pathogens. Without these data, it is not possible to conclusively state whether HLC collectors are at increased health risk or not.

### Recommendations

When determining whether HLC should be replaced with alternative trapping tools, National Malaria Control Programmes (NMCPs) should consider key data needs and select collection tools based on these priorities. A recent publication [63] was developed to help guide decision-makers on how to select appropriate mosquito collection tools for malaria programme needs. To determine equivalency between HLC and alternative traps, two one-sided test analyses should be conducted. If equivalency is determined, no conversion factor would be needed. A major limitation in this approach is that the trap comparisons are based on the total number of *Anopheles* collected/night, and accurately estimating human biting risk remains a challenge. Alternative methods may collect more *Anopheles* than HLC, but this does not mean that they more accurately estimate biting risk. Therefore, if alternative approaches are used to replace HLC, correction factors may be necessary to estimate biting risk for the calculation of EIR. However, a precise conversion factor for metrics such as EIR may not be necessary. While EIR is a very valuable entomological indicator, these values are dynamic and absolute EIR numbers may not be necessary when a relative EIR could suffice to inform vector control decisions. Future work testing for equivalency will provide additional information.

Additionally, to further understand the malaria vector landscape, a metric could be developed to show how HLC and alternative traps perform in the context of vector control interventions.

For this study, the number of *Anopheles* spp. collected per trap per night was used since this was the standard metric represented in the literature when comparing traps to HLC. Although this is the standard, it is not necessarily the best approach. Not all *Anopheles* spp. are malaria vectors, and without a question driven approach to identify which traps are best for certain species, it is not possible to determine which trap will be best in certain situations. A question driven and resource directed approach to trapping mosquitoes for malaria surveillance is necessary. An entomological surveillance planning tool (ESPT) helps guide decision-makers in deciding which trap is best for specific questions related to malaria vector control [63]. This tool may also be used to determine which trapping methods can or should be com- pared in future studies. It is important to note that one major advantage of HLC is that they provide the ability to understand the specific location and biting times of human-seeking mosquitoes. To date, alternative traps cannot reliably replicate this. If the question driven approach is asking when and where mosquitoes bite, there may not be a fully suitable alternative to HLC. There also needs to be a way to minimize heterogeneity. Moving forward, studies should be designed considering target species and trap types. For example, there may be one group or trap type that collects more total numbers of *An. gambiae s.l.*, such as the light trap (Table 6), which does not perform as well for *An. funestus s.l.*

Meta-regression methods show that a linear combination of these variables can explain over 55% of the statistical heterogeneity present. Future work should go towards addressing the questions of trap comparisons to HLC should use standardized and modified methods to help address the remaining heterogeneity. Standardization of methods for future meta-analytic work should account for these variables. Future studies should address heterogeneity variables and publication bias by focusing on questions that address trap types and species outcome. Results should be published regardless of whether findings indicate alternative traps perform better than HLC. When deciding on a collection tool, multiple traps should be used to determine which traps are ideal for specific species. Future studies should report results whether they are capturing more or fewer mosquitoes than HLC along with a corresponding variance metric. Reporting these data will allow for a more robust meta-analysis; as such standardized reporting is necessary for robustness. Temporal analysis of when the mosquitoes were captured was purposely left out of this study because of the lack of standardization of reporting make it impossible to synthesize. Standardization techniques in this area could add another moderator to control for heterogeneity and account for the effects of seasonal effects.

## Conclusions

The results of the meta-regression show that a large percentage of the heterogeneity present in the analysis comes from variations of traps, locations, and species collected. There is not a consensus among publications in the field over whether a specific trap can be used as a “magic bullet” alternative to HLC. Even so, the high between-study heterogeneity and publication bias cannot be ignored. Instead, research on alternative traps should be conducted by performing question-driven studies to address which traps are best for which species. If programmes want to examine *Anopheles* spp. diversity in an area, different trapping tools may be necessary than for programmes that are just interested in a specific vector, such as *An. gambiae s.l.*, or have a specific bionomic question. Rather than aiming to determine which alternative trap can replace HLC, the goal should instead be to identify the optimal trapping tool for question-driven collections needed to inform decisions about appropriate malaria control interventions or for basic research. A baseline assessment of mosquito collection tools relative to HLC in specific locations could be conducted to determine the best tools in specific contexts in response to indicator-driven questions by using the ESPT tool and evaluating results at regular intervals to determine representativeness [63]. In addition, very few studies evaluating collection tools when compared to HLC describe the vector control context and landscape in which the study is being conducted. For example, conducting a study in a context where a vector control tool such as mass distribution of ITNs is used is likely to influence mosquito biting and resting behaviour and the resulting entomological indicators compiled by mosquito collection tools. Under this framework, future meta-analyses could better characterize the landscape of malaria vector behaviour by reducing between-study heterogeneity, allowing for recommendations for malaria vector control interventions that are tailored to local vector ecology.

## Data Availability

The datasets compiled, used, and/or analysed during the current study are available from the corresponding author on request. R scripts used for analysis are available in the malaria meta analysis repository, https://github.com/JordanEckert/malariametaanalysis.

## Appendix

### Discussion on trap category

Initial results did not include moderator analysis results using the trap ‘Category’ variable. This is because classification for each trap is highly subjective; there was a grey area where traps would fall but under new categorization that would not be an issue. A more formal, rigorous definition within the academic community would allow trap category to be used in further analysis.

When examining trap category as a moderator there is still heterogeneity, and no trap type showed a significant difference in *Anopheles* collected per night com- pared to outdoor HLC. When examining sub trap types, there were 25 studies that used biological traps and 22 of those were human-baited alternative traps. Although there is high heterogeneity, there is evidence that human-baited alternative traps capture significantly more *Anopheles* on average per night than HLC (*g* = − 0*.*3940). Multimodel inference added ‘Category’ and the interaction between ‘Category’ and ‘Species’ to the regression model, but the *R*^2^ value decreased slightly. It is highly likely that there is a significant correlation between ‘Type’ and ‘Category’; this is one possible explanation as to why the results from adding the new variables do not improve the heterogeneity explained. When analysis was performed on the *An. gambiae* and *An. funestus* subsets, neither subset offered statistically significant results. However, many categories in both the subsets lack the necessary studies synthesized for statistical power.

## Abbreviations

HLCs: Human landing catches
ESPT: Entomological surveillance planning tool
